# Particle Swarm Optimization and Modular Multilevel Converter Communication in Electrical Applications with Machine Learning Algorithm

**DOI:** 10.1155/2022/8516928

**Published:** 2022-06-09

**Authors:** Shoaib Kamal, Farrukh Sayeed, Tariq Ahamed Ahanger, Chatti Subbalakshmi, R. Kalidoss, Nilu Singh, Stephen Jeswinde Nuagah

**Affiliations:** ^1^Department of Electronics and Communication Engineering, MVJ College of Engineering, Bengaluru, Karnataka, India; ^2^Department of Electrical and Electronics Engineering, ACE College of Engineering, Trivandrum, Kerala, India; ^3^College of Computer Engineering and Sciences, Prince Sattam Bin Abdulaziz University, Al-Kharj, Saudi Arabia; ^4^Department of Computer Science & Engineering, Guru Nanak Institutions Technical Campus, Hyderabad, Telangana, India; ^5^Sri Sivasubramaniya Nadar College of Engineering, Chennai, India; ^6^Department of Computer Science and Engineering, Koneru Lakshmaiah Education Foundation, Vaddeswaram-522502, Guntur, Andhra Pradesh, India; ^7^Department of Electrical Engineering, Tamale Technical University, Tamale, Ghana

## Abstract

As a result of their natural capacity to recover harmonic current and reactive power from alternating current sources, power electronic devices utilized in conjunction with nonlinear loads have the potential to generate significant harmonic problems within the power system when employed in this way. When this occurs, voltage instability occurs, which must be avoided in order to maintain the consistency and dependability of the power system's power flow. With this approach, the series controller has been replaced by a multilevel modular controller in order to improve power handling capability and achieve higher modular levels with minimal distortions. The shunt compensator is the most effective way to achieve an extremely protected energy system as well as righteous steadiness in electric potential difference under a variety of load constraints. The DQ thesis is employed in this proposed converter to separate the harmonic components by establishing reference frame current, which is accomplished by machine learning techniques. As part of the constant mode operation, the PI controller contributes to maintaining the direct current-potential difference, which is given to the PWM generator. Optimization of the values of K *p* and K *i* is accomplished by the use of particle swarm optimization (PSO). The construction of this power system simulation model has been made feasible by the use of time-fluctuating characteristics modeling and the MATLAB programming environment. The new (unified power flow controller) UPFC research that has been made available is persuasive in its capacity to reduce distortions and watt-less power components while simultaneously enhancing efficiency and reducing costs.

## 1. Introduction

**Figure 1:**
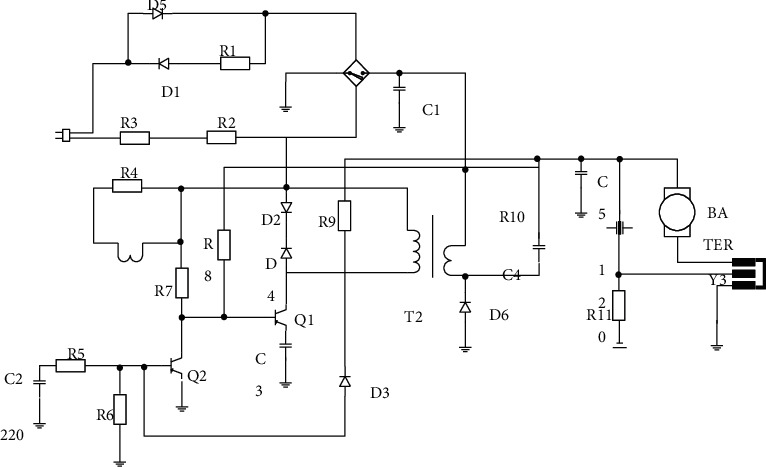
Proposed system circuit diagram.

Power electronics are surpassing mechanical systems in a broad variety of industrial applications, and this trend is expected to continue. It is possible to employ power electronics in conjunction with nonlinear loads to inject harmonic current into an alternating current transmission system, therefore increasing the overall reactive power of the transmission system. [1] The proposed study is focused on minimizing source current harmonic distortions in order to attenuate source current harmonic distortions with high voltage strength under a range of load scenarios. Many researchers examined power flow control strategies, and they used advanced algorithms in conjunction with UPFC to increase the efficiency with which energy flux regulation was applied [2]. Improved energy flux convergence efficiency was the ultimate aim of this project's implementation. [3–5]. It is critical to include some kind of compensation into the system in order to increase the energy transfer capacity and overall stability of the system. In the present study, the issue with the power quality system is addressed by employing a shunt active power filter to fix the situation, which is a novel approach. Only source current compensation may be accomplished via the usage of this filter; no other compensation can be accomplished. Because of the usage of a voltage source inverter, voltage compensation is not feasible with this filter, and high-voltage stress is formed in the shunt filter as a consequence of the use of this filter [6]. In order to cope with this specific circumstance, a static synchronous compensator is used. It may be possible to reduce the voltage stress that has accumulated by using an external DC source. Due to the inability to do harmonic correction using STATCOM, the stability of the system is weakened as a consequence of utilizing this technology. [7–10]. Prior to converting to STATCOM, they employed a static VAR compensator, which is often used in transmission applications where voltage control takes place at weak points in the electrical power system network. Providing reactive power when low voltage is delivered to heavy loads and absorbing reactive power where high voltage is applied to light loads, as well as minimizing line reactance and reactive power consumption, may be used to remedy the problem. [1, 7, 11]. The “unified power flow controller” is a flexible alternating current transference mechanism that will be used for the purposes of this proposed research. Because of its many different characteristics, this technique has a wide range of applications in modern power systems. UPFC combines the features of both STATCOM and SSSC into a single solution that is easy to use [12]. The two voltage source inverters (VSI) that are connected to the distribution line via series and shunt transformers, which are coupled in series and shunt, respectively, are referred to as VSI. The existence of a general direct current-potential difference, which includes the storage capacity [13], makes it possible to integrate all of them. The University of Pittsburgh's research is currently concentrating on system modeling and enhancing the dynamic execution of the grid, both of which are critical parts of grid operation and management. If a fault develops in the transmission line, the fault current is immediately incorporated into the series component of the UPFC, jeopardizing the safety of the UPFC circuit. Additionally, because of the significant discrepancy between the higher and lower voltages [14–18], it has the drawback of taking an excessive amount of time to re-engage in operation, resulting in a delay in re-engaging in operation. Rather than using a series controller, the modular multilevel converter is used in this case [19] (Figure 1). When used in usual settings, it performs in the same way that a conventional converter would. Whenever a flaw is found, high impedance is automatically injected into the system via the current limiting mode in order to preserve the system's normal operating state [13]. Industrial high voltage and higher power applications are successfully penetrated, and output harmonics are steady and low in frequency, paving the way for future advancements in the field. Cross-coupling techniques based on the DQ-axis theory may be used to provide rapid response while also limiting interference between the original and reactive energy fluxes in the same system, allowing for a more efficient operation. The use of quadrature axis voltage and direct axis voltage, respectively, in this control approach enables the adjustment of actual and reactive power to be carried out separately. In order to manage the capacitance voltage and bus voltage of the UPFC, two different axis frameworks are employed, which are both used to manipulate the capacitor voltage [20]. The direct axis framework and the quadrature axis framework are both used to manipulate the capacitor voltage. Once this measurement has been done along the d-q dimensions and compared to the original value, it is sent on to the processing of a PI controller for further processing. The PI controller assigned to this proposed research is responsible for maintaining a constant DC-link voltage; nevertheless, the fuzzy logic-based control system is employed in a number of ways that are prevalent in the industry. This control system stability factor makes a major contribution to the overall stability of the control system in the area of stability. When precise systems are required, tuning a fuzzy system takes an inordinate amount of effort [21]; therefore, it may be essential to utilize a strict conventional design when precise systems are required. Because of the excellent stability and capacity to alleviate the maximum overshoot problem shown by the proposed PI controller [21, 22], it is a good solution for a wide range of applications. It is then sent on to the PWM generator once the voltage stability has been stabilized and adjusted. Using the DQ-axis theory, the desired amplitude and angle (phase difference) of the compensator can be obtained. Then, using a three-phase inverter, the DC electric potential difference can be converted to an alternating current, and the values can be optimized using particle swarm optimization to achieve the desired results.

The following is the sequence in which the parts of the planned work will be presented: After a brief introduction, Section 2 presents the proposed methodology; Section 3 elaborates on the intended work; Section 4 includes the test results; and Section 5 puts the project to a close.

## 2. Proposed Methodology

It is a combination of static synchronous series converter (SSSC) and static synchronous converter (SSC) technology (STATCOM). A DC-link voltage connects the various parts of the system. During the implementation of the suggested technique, an innovative unified power flow controller-based modular multilevel controller is used. In transmission lines, UPFC is employed because of its diverse functions, which include the ability to manage both real and relative energy [23]. When a failure occurs in the power grid, in order to preserve the stability of the grid and to reduce the amount of transitory components produced by the generator, the UPFC provides real and relative power into the system promptly. With the help of a series transformer, the serial compensator and transmission line may be linked in a serial fashion. The series converter converts the DC potential difference from the alternating current (AC) that is collected in the condenser. When the conversion from alternating current to direct current occurs, a large amount of ripple occurs. By incorporating MMC into the proposed methodology, ripples can be reduced, and the shunt converter has the capability of manipulating the electric potential difference of the DC link and the electric potential difference of the bus in which it is bounded, as well as converting the accumulated DC to three-phase alternating voltage. Through the use of a transformer [24], the voltage is provided to the nonlinear load. In order to generate PWM pulses, a hysteresis current controller monitors the difference between the actual current and a reference current in order to calculate the stable load impedance independent current for the load being controlled. A PI controller is used to maintain a constant electric potential difference in the DC link while also suppressing the occurrence of producing harmonics. The electric potential difference between the capacitors existing in DC is measured and analogized with steady voltage in the PI controller [25]. The input to the PI controller is the fault that was discovered, and the output is the maximum value that the wave reaches above the stable value of the current. (S) = K *p* + K *i*/S is the formula for calculating the TFs of the proportional-integral controller. This is accomplished by employing the iterative method PSO to modify the proportional gain, K *p*, and the integral gain, K *i*. The next parts will provide a more in-depth analysis of the situation.

### 2.1. MMC Development Model

**Figure 2:**
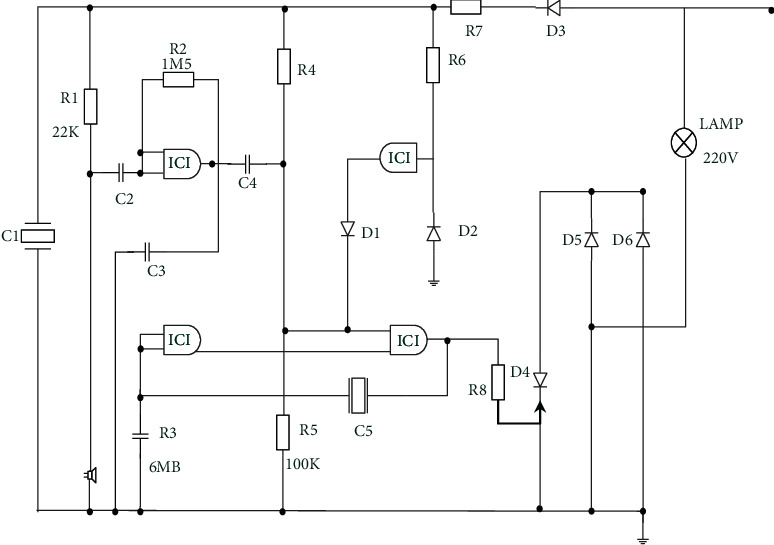
Design of series converter based on MMC.

The topological structure of the MMC is seen in Figure 2. This type is divided into three stages, during which the top and lowermost portions of the bridge are connected to the alternating current line through the arm reactor. The properties of the arm reactor are quite similar to those of the inductor *L* and resistor *R* s, which are also inductive components. The alternating current in the grid is denoted by the symbol *i* si, whereas *i* pi and *i* ni describe the phases of the upper and lower bridge arm currents, respectively, and *i* dc symbolizes the direct current.

As seen in Figure 2, the topological structure of the MMC is visible. There are three steps in the construction of this sort of bridge, each of which involves connecting the top and bottom components of the bridge to the alternating current line through the arm reactor. As with the inductor *L* and resistor *R* s, which are both inductive components, the characteristics of the arm reactor are remarkably similar to those of the arm reactor. The alternating current in the grid is represented by the symbol *i* si, while the phases of the upper and lower bridge arm currents are represented by the symbols *i* pi and *i* ni, respectively, and the direct current is represented by the symbol *i* dc.

The switching representation of the upper- and lower-bunk arms of MMC can be obtained by making use of Kirchhoff's voltage law. It is given as follows:

(1) $$\begin{array}{c} \frac{d{\bar{i_{\text{pi}}}}_{\text{Ts}}}{\text{d}t}=\frac{1}{L}\left(-{\bar{u_{1\Delta \text{ dsi}}}}_{\text{Ts}}+d_{\text{pi}}\;N\;{\bar{u_{\text{smp}}}}_{\text{Ts}}-\;R_{s}{\bar{i_{\text{pi}}}}_{\text{Ts}}\right),\\ \frac{d{\bar{u_{\text{smp}}}}_{\text{Ts}}}{\text{d}t}=\frac{1}{C}\left(-d_{\text{pi}}{\bar{i_{\text{pi}}}}_{\text{Ts}}-\;\frac{{\bar{u_{\text{smp}}}}_{\text{Ts}}}{R_{p}}\right),\\ \frac{d{\bar{i_{\text{ni}}}}_{\text{Ts}}}{\text{d}t}=\frac{1}{L}\left({\bar{u_{2\Delta \text{ dsi}}}}_{\text{Ts}}+d_{ni}\;N\;{\bar{u_{\text{smn}}}}_{\text{Ts}}\;-\;R_{s}{\bar{i_{\text{ni}}}}_{\text{Ts}}\right),\\ \frac{d{\bar{u_{\text{smn}}}}_{\text{Ts}}}{\text{d}t}=\frac{1}{C}\left(d_{\text{ni}}{\bar{i_{\text{ni}}}}_{\text{Ts}}-\;\frac{{\bar{u_{\text{smn}}}}_{\text{Ts}}}{R_{n}}\right). \end{array}$$

Consider *R*_*p*_=*R*_*n*_=*R* and *U*_smp_=*U*_sm_. By computing equations 1 and 2, we get

(2) $$\begin{array}{c} \frac{d{\bar{u_{\text{sm}}}}_{Ts}}{\text{d}t}=\frac{1}{2C}\left(\left.d_{\text{ni}}|{\bar{i_{\text{ni}}}}_{\text{Ts}}|-|d_{\text{pi}}|{\bar{i_{\text{pi}}}}_{\text{Ts}}\right)-\;\frac{{\bar{u_{\text{sm}}}}_{\text{Ts}}}{\text{RC}}\right),\\ \frac{d{\bar{i_{\text{si}}}}_{\text{Ts}}}{\text{d}t}=\frac{1}{L}\left(2{\bar{u_{\text{si}}}}_{\text{Ts}}+\;N\left(d_{\text{ni}}-d_{\text{pi}}\right)\;{\bar{u_{\text{sm}}}}_{\text{Ts}}\;-\;R_{s}{\bar{i_{\text{si}}}}_{\text{Ts}}\right). \end{array}$$

where *i*_si_ is indicated as *i*_si_+*i*_ni_ and *U*_si_ is represented as *U*_si_+*U*_ni_.

The gross resulting voltage of the top and bottom arm in the subcomponent is given as follows:

(3) $$\begin{array}{c} {\text{Nd}}_{\text{pi}}{\bar{u_{\text{sm}}}}_{\text{Ts}}={\bar{u_{\text{dc}/2}}}_{\text{Ts}}-{\bar{u_{\text{oi}}}}_{\text{Ts}},\\ {\text{Nd}}_{\text{ni}}{\bar{u_{\text{sm}}}}_{\text{Ts}}={\bar{u_{\text{dc}/2}}}_{\text{Ts}}-{\bar{u_{\text{oi}}}}_{\text{Ts}}. \end{array}$$

At the time of modeling stage, eliminate the reactive voltage, *U*_dc_ ≈ NU_sm_. Thus, from equation (4), *d*_pi_,  *d*_ni_ are represented in equation (5).

(4) $$\begin{array}{c} d_{\text{pi}}=\frac{1}{2}\left(1-d_{i}\right),\\ d_{\text{ni}}=\frac{1}{2}\left(1-d_{i}\right). \end{array}$$

In each phase, *d*_*i*_ represents the equivalent resulting modulating cycle.

To perform substitution in equation (2), we require the mean switching cycle of MMC in a three-phase model.

(5) $$\begin{array}{c} \frac{d{\bar{i_{\text{si}}}}_{\text{Ts}}}{\text{d}t}=\frac{1}{L}\left(2{\bar{u_{\text{si}}}}_{\text{Ts}}+\text{N}d_{i}{\bar{u_{\text{sm}}}}_{\text{Ts}}\;-\;R_{s}{\bar{i_{\text{si}}}}_{\text{Ts}}\right),\\ \frac{d{\bar{u_{\text{sm}}}}_{\text{Ts}}}{\text{d}t}=\frac{1}{2C}\left(\frac{d_{i}}{2}{\bar{i_{\text{si}}}}_{\text{Ts}}-\frac{i_{\text{dc}}}{3}\;-\;\frac{{\bar{u_{\text{sm}}}}_{\text{Ts}}}{\text{RC}}\right). \end{array}$$

The mean switching cycle of MMC is obtained by making use of park transformation.

(6) $$\begin{array}{c} \frac{L}{2}\frac{d{\bar{i_{\text{sd}}}}_{\text{Ts}}}{\text{d}t}+\frac{R_{s}}{2}{\bar{i_{\text{sd}}}}_{\text{Ts}}={\bar{u_{\text{sd}}}}_{\text{Ts}}+\frac{\omega L}{2}{\bar{i_{\text{sq}}}}_{\text{Ts}}-\frac{\text{N}{\bar{u_{\text{sm}}}}_{\text{Ts}}.d_{d}}{2},\\ \frac{L}{2}\frac{d{\bar{i_{\text{sq}}}}_{\text{Ts}}}{\text{d}t}+\frac{R_{s}}{2}{\bar{i_{\text{sq}}}}_{\text{Ts}}={\bar{u_{\text{sq}}}}_{\text{Ts}}+\frac{\omega L}{2}{\bar{i_{\text{sq}}}}_{\text{Ts}}-\frac{\text{N}{\bar{u_{\text{sm}}}}_{\text{Ts}}.d_{q}}{2},\\ 6C\frac{\text{d}{\bar{u_{\text{sm}}}}_{\text{Ts}}}{2}=\frac{3}{4}{\bar{\left(d_{d}.i_{\text{sd}}+\;d_{q}.i_{\text{sq}}\right)}}_{\text{Ts}}-i_{dc}-\frac{6{\bar{u_{\text{sm}}}}_{\text{Ts}}}{R}. \end{array}$$

### 2.2. Three-Phase Six-Switch Inverter

**Figure 3:**
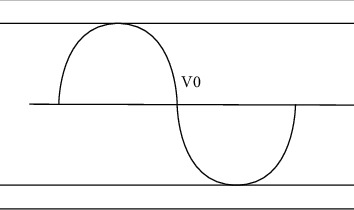
Relationship among the DC, bridge, and AC output voltage of MMC.

**Figure 4:**
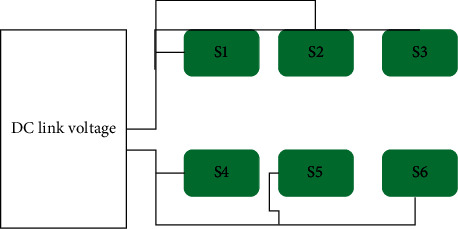
Three-phase six-switch inverter.

**Figure 5:**
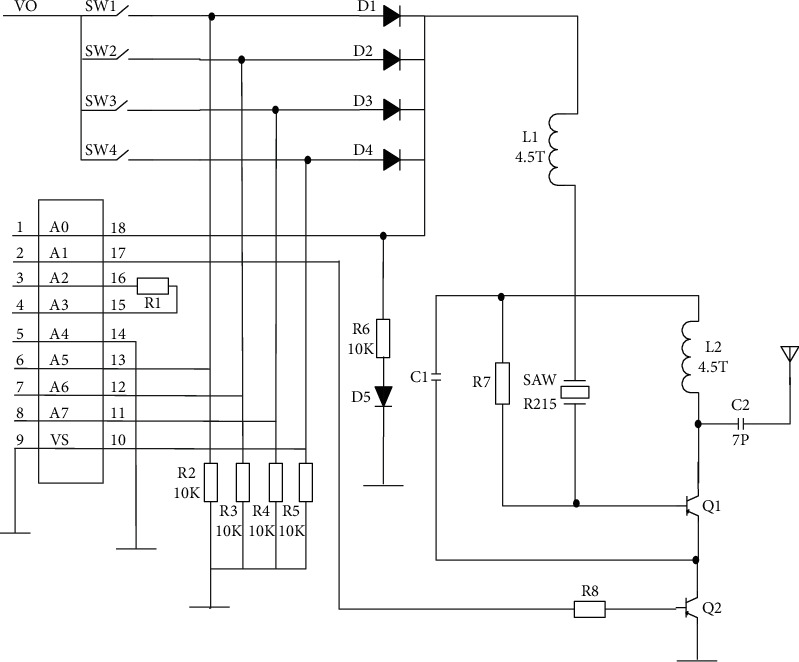
DQ theory modeling for UPFC system.

The drawing of the three-phase six-switch inverter shown in Figure 3 shows its general layout. This power inverter comprises two legs, each of which has three power switches included into it. In addition to this, three sources have been added to the DC link for your convenience [26]. In order to accommodate the three-phased alternating current demands, two phases are included into the two inverter legs, and the remaining phase is fed into the DC source [27]. In order to minimize the number of switches and make them more effective, it is necessary to pair up the middle switches, which are placed in the inverter legs, with alternating current loads [28]. This inverter is capable of performing two different modes of operation. Modes with variable frequencies and modes with a constant frequency are the two alternatives available to you. In the shifting frequency mode, both the frequency and the amplitude of the changes may be adjusted; however, in the stable frequency mode, the frequencies remain the same, but the magnitude of the changes fluctuates, as shown in Figures 4 and 5. [29] as shown in Figures 4 and 5.

### 2.3. UPFC System–DQ Theory Model

Three-phase AC voltage has been partitioned into zero, positive, and negative sequence elements.

(7) $$\begin{array}{c} \left[\begin{array}{c} V_{sa}\\ V_{sb}\\ V_{sc} \end{array}\right]=V_{a}\left[\begin{array}{c} \cos\left(\omega t+\varphi_{0}\right)\\ \cos\left(\omega t+\varphi_{0}\right)\\ \cos\left(\omega t+\varphi_{0}\right) \end{array}\right]\;+V_{b}\left[\begin{array}{c} \cos\left(\omega t+\varphi_{1}\right)\\ \cos\left(\omega t-\frac{2\pi }{3}+\varphi_{1}\right)\\ \cos\left(\omega t+\frac{2\pi }{3}+\varphi_{1}\right) \end{array}\right]+V_{\text{c}}\left[\begin{array}{c} \cos\left(\omega \text{t}+\varphi_{2}\right)\\ \cos\left(\omega \text{t}+\frac{2\pi }{3}+\varphi_{2}\right)\\ \cos\left(\omega \text{t}-\frac{2\pi }{3}+\varphi_{2}\right) \end{array}\right], \end{array}$$

where *V*_sa_,  *V*_sb_,  *V*_*s*_ are represented as AC voltage of three-phase and *V*_*a*_,  *V*_*b*_,  *V*_*c*_ is indicated as zero, positive, and negative voltages, respectively.

(8) $$\begin{array}{c} V_{s}=\left[\begin{array}{c} V_{\text{sa}}\\ V_{\text{sb}}\\ V_{\text{sc}} \end{array}\right]=\left[\begin{array}{c} V_{a0}\\ V_{b0}\\ V_{c0} \end{array}\right]+\left[\begin{array}{c} V_{a1}\\ V_{b1}\\ V_{c1} \end{array}\right]+\left[\begin{array}{c} V_{a2}\\ V_{b2}\\ V_{c2} \end{array}\right]. \end{array}$$

The input current of three-phase is given as follows:

(9) $$\begin{array}{c} I_{s}=\left[\begin{array}{c} I_{\text{sa}}\\ I_{\text{sb}}\\ I_{\text{sc}} \end{array}\right]=\left[\begin{array}{c} V_{a0}\\ V_{b0}\\ V_{c0} \end{array}\right]+\left[\begin{array}{c} V_{a1}\\ V_{b1}\\ V_{c1} \end{array}\right]+\left[\begin{array}{c} V_{a2}\\ V_{b2}\\ V_{c2} \end{array}\right]. \end{array}$$

The estimated power is computed in the following equation:

(10) $$\begin{array}{c} S_{s}=P_{s}+{\text{iQ}}_{s}=V_{s}I_{s}^{*}. \end{array}$$

The general equation of the DQ-axis theory can be written as follows:

(11) $$\begin{array}{c} S_{s012}=S_{l012}+S_{f012}, \end{array}$$

where zero, positive, and negative sequence power is represented as 0, 1, and 2, respectively, and the overall power infused to the grid is evaluated as follows:

(12) $$\begin{array}{c} S_{l012}=S_{s012}+S_{h012},\\ S_{l012}=p_{s012}\left(t\right)+Q_{s012}\left(t\right)+p_{h012}\left(t\right)+Q_{h012}\left(t\right). \end{array}$$

where the parameters *P* and *Q* indicate the real and reactive power of the transmission system, respectively.

(13) $$\begin{array}{c} V_{\alpha \beta 0}=\frac{2}{3}\left[\begin{array}{ccc} 1 & -\frac{1}{2} & -\frac{1}{2}\\ 0 & \frac{\sqrt{3}}{2} & -\frac{\sqrt{3}}{2}\\ \frac{1}{2} & \frac{1}{2} & \frac{1}{2} \end{array}\right]V_{s},\\ I_{\alpha \beta 0}=\frac{2}{3}\left[\begin{array}{ccc} 1 & -\frac{1}{2} & -\frac{1}{2}\\ 0 & \frac{\sqrt{3}}{2} & -\frac{\sqrt{3}}{2}\\ \frac{1}{2} & \frac{1}{2} & \frac{1}{2} \end{array}\right]I_{s}. \end{array}$$

The voltage and current of the three-phase input are transformed to *αβ*0 parameter by making use of Clarke transformation.

The reference signals are derived from the source potential and current by applying the park and Clarke transformation to the source potential and current. The conversion of the reference signal to the actual value is accomplished via the employment of hysteresis comparator technology [30]. For example, the PWM pulses that are fed into the S/S converter are the result of the hysteresis current controller being activated. The PI controller is responsible for maintaining a consistent DC-link voltage [31].

It is one of the responsibilities of the DC electric potential difference regulator to track the real voltage across the capacitor, and the error is calculated by subtracting the difference between the set voltage and the actual voltage across the capacitor. The calculated fault is sent as source data into the PI controller, which then computes the fluctuations in the DC potential that are present [32]. This is accomplished by retrieving the result of the PI controller and using it as a reference for the real composition of ion flow recovered by the UPFC system. An analysis of the relationship between these two reference values and the actual quantities is carried out in a cross-coupled controller. When the capacitor voltage is traced and fostered in a stable state [33], the PI controller is used to accomplish this. The following is the formula for computing the transfer function of the PI controller [34].

In this suggested technique, particle swarm optimization is used to determine the ideal proportional and integral gain values for each parameter. PSO is the ideal approach for addressing a nonlinear optimization issue since it is fast and efficient. It is the optimization strategy that imitates the community properties of many kinds of leaves [31]. It is a technique for optimizing the performance of computer programmes. This approach is based on the number of fragments used in exploring the inner region of a multidimensional space, and it is very fast [35]. The primary goal of this iterative approach is to improve the performance of the PI controller's parameters (K *p* and K i). In the exploration region, it seeks to find the most optimal resolution possible. By simulating the particle, PSO finds the optimal solution in a search space that has been specified. In this particle swarm optimization approach, a population of the swarm is started with random velocities *Vi* and positions S *i*, and the swarm is then allowed to optimize. As a starting point, each particle of the population is randomly distributed over the whole search space [36]. Then, with guidance from the population criteria, it modifies its velocities in accordance with its own flying experience and the flying experience of their colleague [37]. Each particle keeps track of the best position it has recovered thus far, as well as the global best position that has been achieved by any number of particles.

Where *C*_1_ and *C*_2_ are positive constants and *R*_1_,  *R*_2_ are random numbers that lie in between the range of 0–1. The parameter *W* is represented as weight that enhances the overall effectiveness of PSO.

In parallel with the growth in the number of power electronic converter applications across many sectors, so too are the issues connected with their use. Multilevel inverters (MLIs) are one of the most important technologies utilized in renewable energy and electrification, and its dependability and fault ride-through capabilities are highly desired characteristics to have [38]. It is necessary to have fault tolerance against switch open-circuit faults in power electronics systems when using a large number of semiconductor components, which are the leading cause of failures in power electronics systems, especially in remote applications with significant maintenance penalties or in safety-critical operations. In this research, we offer a fault-tolerant asymmetric reduced device count multilevel inverter technology that produces an 11-level output under healthy circumstances and is capable of running even when an open-circuit fault occurs in any switch. An NLC-based pulse width modulation scheme is established, and the scheme is modified postfault in order to maintain acceptable power quality throughout the system's lifetime [22]. The structure is subjected to a reliability study in order to determine the advantages of using fault tolerance. The topology is compared to a number of fault-tolerant topologies that have been studied recently in the literature. As an added bonus, a machine learning classification issue utilizing decision trees is suggested to be used in conjunction with artificial intelligence (AI) to find and correct faults. The fault detection approach is effective in finding the location of faults while using little processing resources and achieving a satisfactory level of accuracy [39].

**Figure 6:**
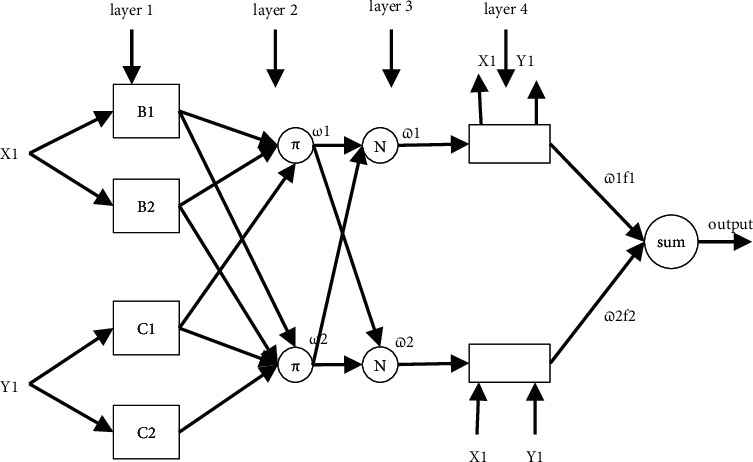
Flowchart for neural network.

The flowchart of the proposed work is added in Figure 6. They were created with a single hidden layer, the gradient descent with momentum (GDM) method as the learning rule, TanhAxon as the transfer function for the hidden layer, and BiasAxon as the transfer function for the output layer and with a single hidden layer. A cross-validation approach known as leave-one-out cross-validation was used to train the neural networks in this study [21]. As a result of using this strategy, the data set is divided into two kinds of information: training data and validation data. There is an alternate approach for determining the correctness of a model that does not need the development of additional data to be used. A network is trained several times in this scenario; however, for each run, different bits of data are omitted from the network. When evaluating the durability of a model on small datasets, it is quite useful to use this training strategy [40]. This section presents the results of dividing the 24 data points into 6 (*N* = 6) groups of about comparable size. Following that, we trained the net *N* times, each time leaving out one of the subgroups from training but only utilizing the omitted subset to determine error criteria. After that, we trained the net *N* times more (as opposed to using the whole training set). The data collection used to train the ANN models was provided by the researchers. Furthermore, the test data for all models is the same, allowing for a comparison of the performance of the different models [41].

## 3. Results and Discussion

With the help of MATLAB simulation, the developed procedure is performed alongside the IGBT device, and the shunt and series converter is developed. This system is carried out with 100 V AC which is utilised to contribute to the varying load. As the input power exhibits a weighted distortion, using the proposed system, the distortion can be minimized as well as maintains stability in the power system.

**Figure 7:**
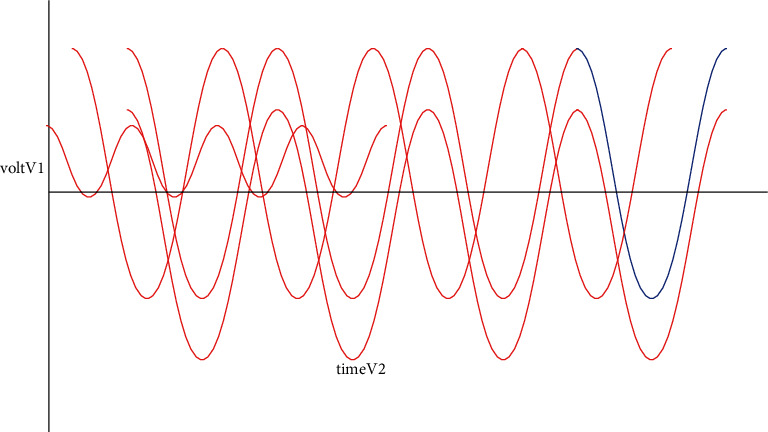
Input AC voltage waveform.

Figure 7, depicts the three-phased AC potential difference waveform. Under various voltage circumstances, for inspecting, the actual electric potential difference has plunged between 0.4 and 0.6 sec. Under this condition, for insertion of the reimbursement of voltage, the proposed technique nurtures voltage stability.

**Figure 8:**
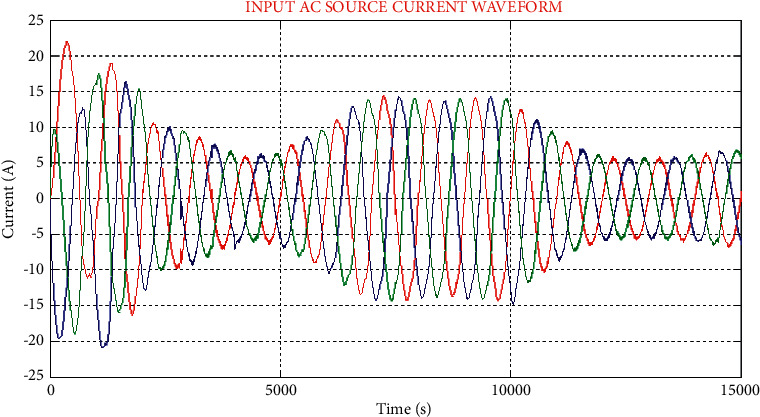
Waveform of input AC source current.

In Figure 8, the waveform of AC source current is displayed, which shows maximum order harmonics, but by making use of the proposed UPFC system, the occurred harmonics are minimized in the source current.

**Figure 9:**
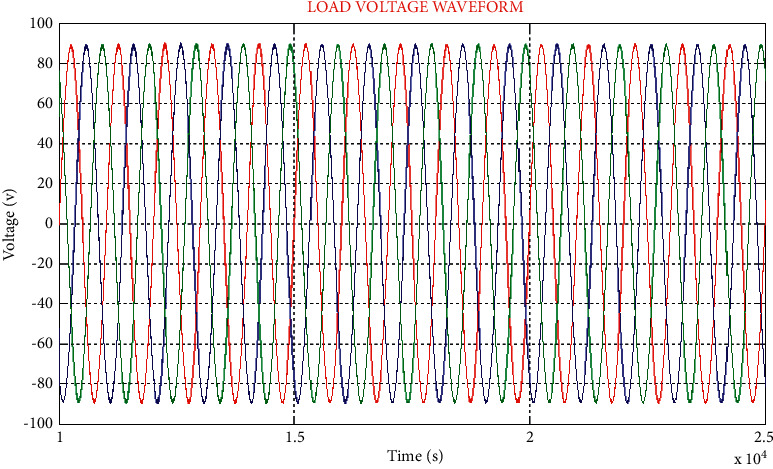
Waveform of load voltage.

To the nonlinear load, three-phase AC voltage is delivered. The UPFC regulates the voltage in a stable mode. After achieving stability, the stable voltage is delivered into the system as a sufficient constant electric potential difference is retrieved from the condenser of the DC link, as shown in the Figure 9.

In the sinusoidal load current's waveforms are illustrated. Distortion is reduced due to which the waveform is succeeded by the DC voltage unlocking converter in both the source and load sides.

DC-link voltage waveform is represented in by utilizing PI controller, the series converter regulates the DC voltage in a stable mode. By this voltage, the shunt converter generates the steady potential difference function for the load.

**Figure 10:**
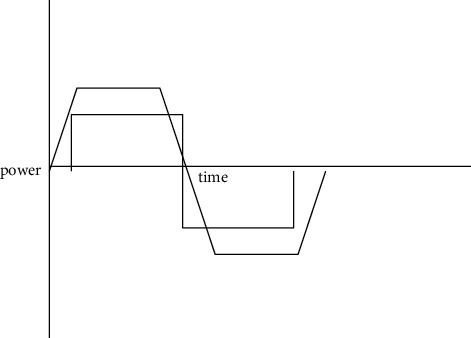
Source side and load side power waveform.

The UPFC system has a good capability to minimize the reactive power and to increase the real power on both sides. It is represented in Figure 10.

The reactive energy at the transmission line may be raised with the aid of the nonlinear load present on the line. A PCC absorbs energy at that point, which may then be stored in the condenser of the DC-link [38], which is then used by the MMC converter.

Meanwhile, the shunt converter, which makes use of the shunt transformer, incorporates real power into the power conversion circuit. The MMC and waveforms of the shunt converter are shown in respectively, in the figures.

Evolutionary algorithms have been used to solve a vast variety of engineering problems, and it has been shown that these methods are successful in a wide range of situations. The control parameters, on the other hand, are a major source of concern when utilizing these algorithms since they may have a detrimental influence on the algorithm's performance. Additional concerns include the size of the population, the rate of convergence, and the cost of computation, all of which are critical elements in the success of the approach and are thus quite important. A number of well-known optimization techniques exist, including particle swarm optimization (PSO), genetic algorithms (GA), ant bee Colony (ABC) algorithms, imperialistic competitive algorithms (ICA), and TLBO. These algorithms, as well as others, are subjected to the selective harmonic elimination technique of elimination. This article covers a newly proposed optimization technique that has been further improved, beginning with the teaching-learning approach as a starting point. As a result of the fact that it is free of controlling elements, it is simple to design and easy to control its effectiveness, among other qualities; this algorithm has become a frequent tool for handling most optimization problems.

The influence of the teacher on a student's performance in each area is the core assumption of these algorithms, and this is the basis for their development. In this particular instance, the number of design variables is proportional to the number of participants. In order for students to gain information, it is the teacher's responsibility to do so. A high-quality teacher leads to a more successful student in terms of academic achievement.

## 4. Conclusion

The proposed work has as its primary control objective the regulation of the flow of power via the cable system. The conclusion is that one of the most essential strategies for dealing with the DC bus electric potential difference is to compromise the real power balance on both sides and to expand the DC bus electric potential difference within the boundaries of a safe operating procedure. It is proposed that the operating criteria and power behaviour of extremely dependable MMC-UPFC be investigated; on the basis of this, targeting the various operating circumstances with minimal extent adjustments alongside real/active power flux and the maximum extent and fault in the shunt side converter is targeted. For the series and parallel faces of the MMC-UPFC, an extended powerful model is accepted, and associated handling strategies are presented based on the control target and computational techniques assumed from the control target and computational techniques assumed from the control target and computational techniques. Through the use of simulation, it is possible to put into action situations that would be difficult to control otherwise. When tested under a variety of operating constraints, the experimental findings demonstrate that the voltage of the MMC-DC UPFC's bus is guaranteed to be in the constant mode within a secure range, enabling the user to perceive the system's stable and steady-state performance.

Because of this research, a unique DCDC converters model, based on Bayesian regularization-based artificial neural networks (ANNs) and the random forest approach, has been developed and is being tested. It was discussed how to construct a model for the boost converter, including the data collection and processing methods, as well as the training, testing, and validation stages. It was also discussed how to design a model for the boost converter. When the machine learning-based models were tested, it was observed that they were capable of producing responses that were remarkably comparable to those produced from the simulations. These approaches are projected to be attractive for the implementation of contemporary concepts such as digital twins, circuit automation, and software-defined electrical networks since they enable rapid and cost-effective modeling platforms.

Machine learning-based models are also computationally light and can be implemented on graphics processing units (GPUs) or field-programmable gate arrays (FPGAs), which is an important factor in allowing next-generation edge computing in power converters.

They may also replicate the voltage across (and current through) the switches if a substantial quantity of data is utilized to train the algorithms that are proposed.

This work serves as an introduction to the topic of modeling power converters using machine learning methodologies, which will be covered in further depth in the next paper. Research in this subject will continue to develop the models to better capture different operational scenarios, study alternative machine learning methodologies for better results, and widen the scope of the models to include additional converters in a broader range of applications.

## Data Availability

The data that support the findings of this study are available on request from the corresponding author.
